# Estimating the extent of household contact misclassification with index cases of disease in longitudinal studies using a stochastic simulation model

**DOI:** 10.3402/gha.v6i0.19614

**Published:** 2013-01-24

**Authors:** Tobias Chirwa, Sian Floyd, Paul Fine

**Affiliations:** 1 Division of Epidemiology and Biostatistics, School of Public Health, Faculty of Health Sciences, University of the Witwatersrand, Johannesburg, South Africa; 2 Department of Infectious Disease Epidemiology, Faculty of Epidemiology and Population Health, London School of Hygiene and Tropical Medicine, London, UK

**Keywords:** household, contact, simulation, misclassification, demography, sensitivity, longitudinal studies

## Abstract

**Background:**

Household contact with an index case of an infectious disease is a known risk factor for infection transmission. However, such contact may be underestimated due to the dynamic nature of households, particularly in longitudinal studies. Such studies generally begin with contact defined at a single point in time (‘snap-shot’), leading to contact misclassification for some individuals who actually experienced contact before and after the snapshot.

**Objective:**

To quantify contact misclassification with index cases of disease in households.

**Methods:**

Historical data of 112,026 individuals from 17,889 households from an epidemiological study on leprosy in northern Malawi were used. Individuals were interviewed in the early 1980s and followed up over 5 years. It was possible to trace whether individuals died, changed household within the area, or moved out of the area between the two surveys.

Using a 10% sample of households as the starting population and parameters for demographic and household changes over 5 years, the extent of contact misclassification was estimated through a simulation model of household dynamics, which traced contact with index cases in households over time. The model thereafter compared initial contact status and ‘true’ contact status generated from simulations.

**Results:**

The starting population had 11,401 individuals, 52% female, and 224 (2%) leprosy index cases. Eleven percent of the households had at least one index case resident and 10% (1, 177) of non-case individuals were initial contacts. Sensitivity of initial contact status ranged from 0.52 to 0.74 and varied by age and sex. Sensitivity was low in those aged 20–29 and under 5 years but high in 5- to 14-year-olds. By gender, there were no differences among those aged under 5; females had lower sensitivity among those aged under 20 and higher for those above 30, respectively. Sensitivity was also low in simulations of long incubation periods.

**Conclusion:**

This work demonstrates the implications of changes in households on household contact-associated disease spread, particularly for long durations of follow-up and infections with long incubation periods where earlier unobserved contact is critical.

One way to determine risk of disease in populations or communities is through contact tracing to see whether someone who has had contact with index cases develops the disease. Such contact is often recorded at school, household or familial level – standardised settings which control for the environment or human behavioural factors.

Over decades, households and closed communities have proved to be useful (1–4) for generating knowledge of the communicability of various infections. In these instances, exposure and susceptibility are derived by observing spread within households, where close contact and mixing are easily identified (2, 5–8). If an infected person is present in a household, household members may be at an elevated risk of infection because closeness of contact is likely to be related to dose intensity or degree of infectivity (1, 2), which, in turn, is related to the infection transmission and occurrence of disease.

There have been several studies of infection transmission in households, mainly acute communicable diseases such as measles, influenza, and diphtheria (1–6, 9–13), where index cases of infection are easily identified and incubation periods are short. In contrast, for chronic infections (14–16), it is much harder to derive infection transmission knowledge because of the long and variable incubation period of the disease (17, 18) and the dynamic contact networks over time (6, 7, 19). Although many studies on actual transmission have been carried out and are well documented, very few (5, 6, 19) have looked at the number of the susceptible population that have been missed due to the assumption that the contact's details have remained static (19–22).

Although there are practical challenges, studies have been conducted which have attempted to trace contacts over time. For example, studies of newly diagnosed tuberculosis cases emphasise examining all close contacts at the time of diagnosis (14, 23–25) and retrospective investigation to trace their contact histories (26, 27). However, contact histories of diagnosed cases prior to the time of diagnosis are subject to considerable error, especially so for those who lived in different households compared to the one in which they lived when recorded at the time of diagnosis (2, 15, 24, 26, 27). Furthermore, casual and transient contact with infectious cases in these households, over time, may go unnoticed but could play a significant role in transmission of infection. Models developed on the risk of contact are simplistic and make assumptions that are not informed by direct data sources (6, 7, 19). Therefore, there is considerable scope for misclassification. This necessitates investigations through simulations of household dynamics to provide an understanding of the magnitude of the problem of contact misclassification through dynamic contact networks.

While some studies have acknowledged problems of misclassification and the use of simplistic random mixing assumptions (6, 28), very few have investigated these issues further except through validation studies conducted outside the study populations (6, 21, 23, 24, 29). This study aimed to quantify misclassification of contacts with an index case of disease in households.

## Methods

### Study design and population

This study utilised data from a large epidemiological study of leprosy in Karonga District, Northern Malawi. Details of the methodology, including data management and procedures to ensure good quality data, are explained elsewhere (30, 31). Briefly, the data were collected through two linked population surveys. The first survey (LEP-1) was carried out from early 1979 to 1984 and the second survey (LEP-2) from 1986 to 1989. Individuals were assessed for disease in LEP-2. Individuals were uniquely identified and linked to households in which they resided during LEP-1 and LEP-2. An effort was made in LEP-2 to trace all individuals identified in LEP-1. It was therefore possible to collect socio-demographic data and trace whether individuals died, changed household within the area or moved out of the area between the two surveys.

A household was defined as a group of people living together and acknowledging one person as the head. Information collected on uniquely identified households included geographical location, head of household and household assets. Index cases were defined as individuals diagnosed as having leprosy prior to or at their first examination in the LEP-1 survey. Incident cases were those cases diagnosed only *after* the first examination. Observed contacts were individuals who resided in a household with an index case of leprosy whereas non-contacts were individuals who were assumed to never have had any household contact with an index case of disease.

### Sensitivity of initial contact status

Before describing the stochastic micro-simulation model, we first define sensitivity of initial contact status. The simulation model of household changes traced contact histories of individuals over time. The contact status generated through simulations was considered as the ‘true’ underlying state of household contact status of an individual by the end of follow-up. This study compared initial (observed) contact status at baseline and ‘true’ underlying contact status from simulations and computed *sensitivity* of initial contact status, defined as the proportion of true contacts that were correctly observed as contacts at the start of follow-up.

Due to the long and variable incubation period of diseases such as leprosy and tuberculosis, some incident cases that arise during the follow-up period may be attributable to earlier household contact before the period of study rather than that recognised at baseline or during follow-up. Thus, sensitivity was calculated using two approaches to take into account, separately, household contacts before and during the study.

‘Forward’ sensitivity is appropriate for situations in which the incubation period of disease is relatively short and most contacts leading to disease occur during the study period. ‘Forward’ sensitivity of contact status was thus defined as the proportion of individuals in contact with at least one (index) case at any time during follow-up that were correctly observed as contacts at baseline.

‘Backward’ sensitivity relates to the measure of relevant contact, which occurred in the past, before baseline assessment. ‘Backward’ sensitivity of contact status was defined as the proportion of individuals who were in contact with at least one (index) case prior to the onset of follow up that were correctly recognised as contacts at the start of follow-up.

### Micro-simulation model for household dynamics

A stochastic micro-simulation model of household dynamics, which affect household contact status over a period of time, was developed using extrinsic processes, mainly demographic events (32), model dynamic contact networks, as outlined by Bansal et al. (19). The key demographic events modelled at individual level were: birth, death, marriage, migration into and out of the district, respectively, and individual household change within the district. These events were chosen because they are critical to changes in a population structure and that directly affect household composition, with implications for household contact with index cases of disease.

An initial sample of individuals and their attributes (32) was obtained from the actual LEP population defined by age, sex, and household. Initially, non-case individuals who were resident in households with an index case of disease were considered as contacts whereas those who were resident in non-case households were considered as non-contacts. The demographic events were simulated on an individual and annual basis. Histories of household contact with index cases were tracked through the simulation model and values of sensitivity of initial contact status were calculated based on these histories. The annual probabilities of an individual experiencing change of household and each of the demographic events were derived from either the LEP or census data (Table 1) (33).

**Table 1:** Annual probabilities of demographic events occurring by age and sex, Karonga District, northern Malawi 1979–89

		Age group (in years)								
		
Event	Sex	0	1–4	5–9	10–14	15–19	20–24	25–29	30–44	45 +
Death	Male	0.1523	0.0392	0.0077	0.0047	0.0033	0.0038	0.0069	0.0063	0.0254
	Female	0.125 9	0.0297	0.0071	0.0045	0.0024	0.0062	0.0051	0.0074	0.0226
Change of household	Male	0.0500	0.0500	0.0439	0.0476	0.0836	0.1274	0.0799	0.0321	0.0168
	Female	0.0528	0.0528	0.0558	0.1125	0.1801	0.1079	0.0658	0.0428	0.0347
Out-migration	Male	0.0229	0.0229	0.0204	0.0247	0.0402	0.0535	0.0403	0.0283	0.0075
	Female	0.0252	0.0252	0.0276	0.0321	0.0387	0.0459	0.0228	0.0173	0.0049

**Figure 1:**
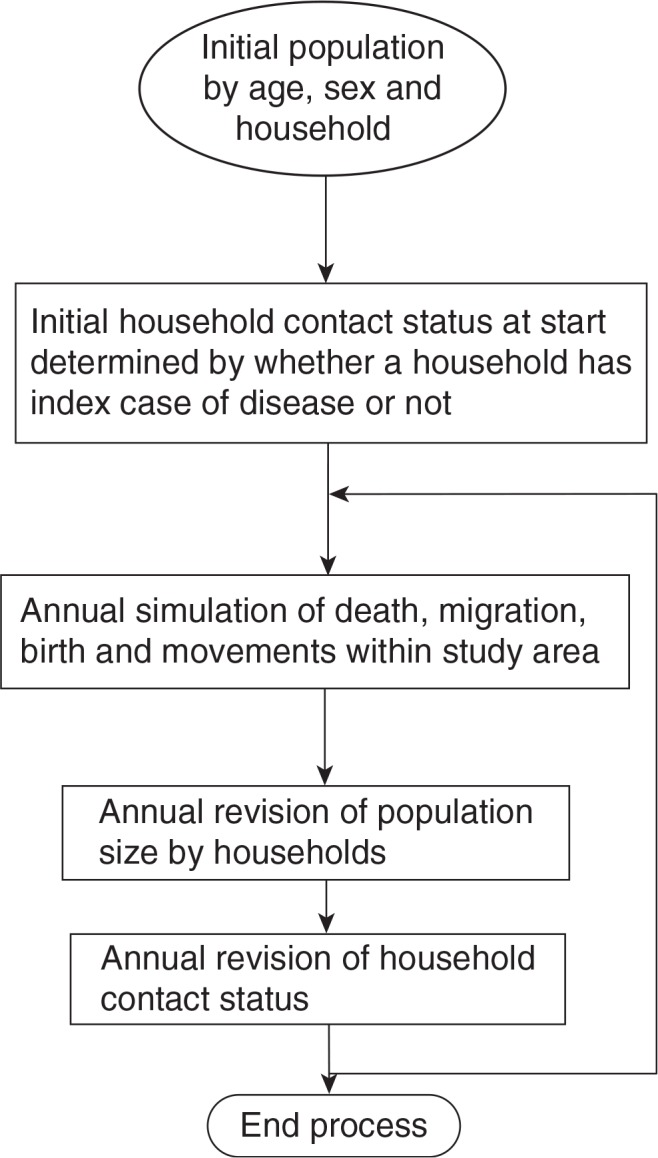
Overview of the stochastic micro-simulation model for household dynamics.

Figure 1 presents an overview of the micro-simulation model. Only key procedures for household contact are presented below, with further details of the specific procedures presented in Appendix A.

#### Change of household

The simulation modelled annual movements of individuals within the study area by checking whether their current household was different compared to the one they resided in the preceding year. If the two households were different, then the model recorded a change in household.

At the start of the simulation, the ‘true’ contact status of an individual was set to either ‘Yes’ if they were already in household contact with an index case at the start of the simulation or ‘No’ if they had no such household contact. The ‘true’ contact status was successively updated on an annual basis during the simulation period. In addition, the initial duration of contact with an index case was set to either 0 if one was not in household contact with a case or one for those already in contact with an index case at the start of the simulation.

If an individual who had been in contact with a case before moved into a case household, the duration of contact was incremented by one. If the individual had not been in contact with a case before only their ‘true’ contact status is changed to ‘Yes’. For all changes in household that were executed by the simulations, the new and previous sizes of households were increased and decreased by 1, respectively.

Furthermore, if an index case of disease moved into a household, the ‘true’ contact status of individuals in that household who were not in contact with a case before was changed and duration of contact was increased by 1. However, if individuals in the new household were already contacts, only the duration of contact was increased.

#### Demographic events

The positions of individuals in a household were categorised as ‘head’, ‘member’, and ‘other’ (visitor, employed worker, renter, or their relatives). A ‘member’ could be a spouse, child, or a relative to the spouse of head or head himself. Allocation of positions to household occupants was carried out to maintain culturally recognised household structures after simulating demographic and other key events, but did not have any implications for determining contact status.

If any individual other than a head of household died, out-migrated or changed household, the individual's record was marked for removal from their current household. If the individual were in a single-person household, that household was dissolved.

A head of household is a key individual and any demographic event associated with a head has implications for household dissolution and further contact with index cases of disease. If a head of household died, out-migrated, or changed household within the district, the oldest ‘member’ of that household aged more than 18 years was assigned as the new head. However, if the oldest ‘member’ was below 18, that household was dissolved and its occupants were randomly allocated to other existing households using the neighbourhood preference approach explained in this article. Such allocation of positions and to households was justified from the LEP data (33).

#### Household allocation procedure

Allocation of households to individuals marked as having either changed households, in-migrated or whose households had been dissolved was carried out on an annual basis. Individuals who changed households were either randomly allocated (with probability 0.05) to newly formed households or to existing households.

The allocation of individuals to existing households was influenced by ‘proximity’ of households. It was assumed (and this was the case in LEP data) that the closer the household serial numbers, the smaller the physical distance between the households and the more likely a movement was to occur between them.

Generation of the destination existing (new) household serial number for individuals who moved was a function of the previous household, based on a standard normal random value and pre-determined constant standard deviation. Explicitly, *H*_*n*_=*H*_0_±Z* σ, where *H*_*n*_ and *H*_0_ are new and previous household serial numbers; *Z* and σ are the standard normal random value and standard deviation, respectively. The choice of standard deviation was such that a large proportion of individuals were allocated to nearby households although some still moved considerable distances.

#### Generation of incident cases

It was inevitable that some initial index cases would be lost to migration and death during the simulation period. Thus, to avoid depletion of ‘index cases’ over time, incident cases were generated based on age and sex incidence rates obtained from previous studies in the same population. The incident cases generated in simulations of long incubation periods were assumed to become infectious at onset of disease and to remain infectious for 3 years.

### Simulation runs

In general, 50 simulations of up to a 5-year period each were run and each simulation run produced ‘forward’ and ‘backward’ sensitivity of contact status values by age and sex. For ‘backward’ sensitivity, the length of observation time prior to the start of study, required to identify the relevant ‘window of opportunity of contact’ was carefully defined since not *all* earlier contact may be relevant for disease that may be observed in the study period.

This was achieved by running a series of simulations of 10-year periods and investigating how varying the length of observation time prior to the start of study affected household contact status misclassification. Fifty simulations of 10-year periods each were run with incubation periods of 5, 7 and 9 years separately. For example, to calculate sensitivity based on an *n*-year incubation period and for a 10-year simulation period, we were interested in contacts arising between *b-n* and *e-n*, where *b* and *e* are beginning and end of the cohort study period, respectively.

The mean sensitivity of contact status was the average over all the simulation runs. The 95% confidence intervals of sensitivity of contact status are calculated assuming normality with standard deviation calculated from the generated sensitivity values. The crude 95% confidence intervals (34) were given by obtaining the 2.5th and 97.5th percentiles of values of sensitivity of initial contact status. Values of the sensitivity have been estimated for different age and sex categories and long incubation period circumstances. The simulations were run using SAS/IML (Macros).

#### Stability of the model

Stability of the model was investigated by comparing structure of the population and household size distribution before and after simulations. The household size, age, and sex distributions were approximately similar (data not shown) reflecting how well the simulation captures the original structure. The minor differences were considered acceptable for the purpose of tracking contact status.

## Results

This section presents results from the simulation model. It includes both forward and backward sensitivity of contact status based on annual household changes and also based on the duration of follow-up. Results from demographic and household dynamics analysis based on these LEP data have been published elsewhere (33, 35).

### Sensitivity of contact status

#### Forward sensitivity based on lower and upper mean rate of household change

Being the most important input parameter for determining misclassification, simulations of 5-year periods each were run separately using the 95% lower and upper confidence limits of the annual rate of change of household (Table 2) to assess the extent of variation in sensitivity. The sensitivity values obtained from these simulations were compared to those obtained using the ‘mean’ annual rate of household change. Only small differences in the ‘forward’ sensitivity of contact status were observed regardless of whether one used the 95% confidence limits or mean. Thus, it was deemed adequate to only investigate sensitivity values based on the mean annual rate of changing household.

**Table 2:** Annual probabilities of change of household (95% confidence intervals), return moves and in-migration by age and sex, Karonga District, Northern Malawi, 1979–89

95% confidence interval for annual probabilities of change of household by age and sex, Karonga District, northern Malawi 1979–89										
Sex										
Male	Lower limit	0.0477	0.0477	0.0417	0.0450	0.0796	0.1208	0.0742	0.0284	0.0127
	Upper limit	0.0523	0.0523	0.0460	0.0502	0.0878	0.1342	0.0853	0.0357	0.0212
Female	Lower limit	0.0504	0.0504	0.0533	0.1081	0.1739	0.1020	0.0621	0.0403	0.0297
	Upper limit	0.0552	0.0552	0.0584	0.1170	0.1866	0.1140	0.0694	0.0478	0.0442
Conditional probabilities of individuals making return (as opposed to forward) moves to households by age and sex, Karonga District, northern Malawi 1979–89										
Male		0.52	0.52	0.52	0.58	0.24	0.33	0.20	0.44	0.75	
Female		0.41	0.41	0.41	0.27	0.18	0.14	0.38	0.21	0.36	
Annual in-migration rates (per person) in Karonga District, Malawi 1986–89										
Male		0.0120	0.0120	0.0192	0.0188	0.0241	0.0315	0.0349	0.0202	0.0068	
Female		0.0115	0.0115	0.0185	0.0246	0.0349	0.0417	0.0436	0.0184	0.0083	

#### Forward sensitivity of contact status

Table 3 presents estimates of the ‘forward’ sensitivity of contact status by age (defined at LEP-1) and sex, with duration of follow-up varying from 1 to 5 years.

**Table 3:** Forward sensitivity of initial contact status by age and sex from 50 simulation runs with different duration of follow-up based on contact with all index cases

	Duration of follow-up (in years)									
	
Age group (in years)	1		2		3		4		5	
				
	Male	Female	Male	Female	Male	Female	Male	Female	Male	Female
0	0.89	0.87	0.79	0.80	0.71	0.71	0.66	0.65	0.62	0.60
1–4	0.93	0.91	0.86	0.83	0.78	0.78	0.70	0.70	0.66	0.66
5–9	0.92	0.92	0.84	0.82	0.81	0.78	0.77	0.72	0.73	0.68
10–14	0.90	0.89	0.85	0.81	0.79	0.73	0.74	0.69	0.72	0.65
15–19	0.87	0.87	0.80	0.78	0.72	0.72	0.64	0.64	0.62	0.58
20–24	0.87	0.83	0.77	0.75	0.69	0.69	0.66	0.66	0.59	0.61
25–29	0.86	0.89	0.77	0.79	0.69	0.67	0.60	0.54	0.56	0.52
30–44	0.87	0.90	0.76	0.81	0.69	0.76	0.64	0.71	0.62	0.67
45+	0.90	0.92	0.85	0.87	0.79	0.82	0.74	0.77	0.70	0.74

As expected, the sensitivity declined with duration of follow-up. Sensitivity values for a 1-year period of follow-up were over 85% whereas those for a 5-year period ranged between 50 and 75%.

The age and sex distribution of sensitivity values for the 5-year duration of follow-up were the same under 50 and 100 simulation runs of the model. The sensitivity values showed no apparent sex differences for children aged less than 5. The distribution of the forward sensitivity peaked at 73 and 68% in boys and girls aged 5–9 years, respectively. The values were lower among females than males in age groups 5–19 and were lowest in the 25–29 age groups in both males and females (56 and 52%, respectively). Household contact misclassification decreased with age for individuals over 30, with greater misclassification for males than females.

#### Crude 95% confidence intervals for ‘forward’ sensitivity

The sources of uncertainty in sensitivity of contact status may be due not only to variation in change of household but also to stochastic variability in the simulations. The latter reflects our uncertainty about the relation between the contact status and demographic events of interests. In that regard, the crude 95% confidence limits of sensitivity of contact status from the simulation model capture this stochastic variability.

**Figure 2:**
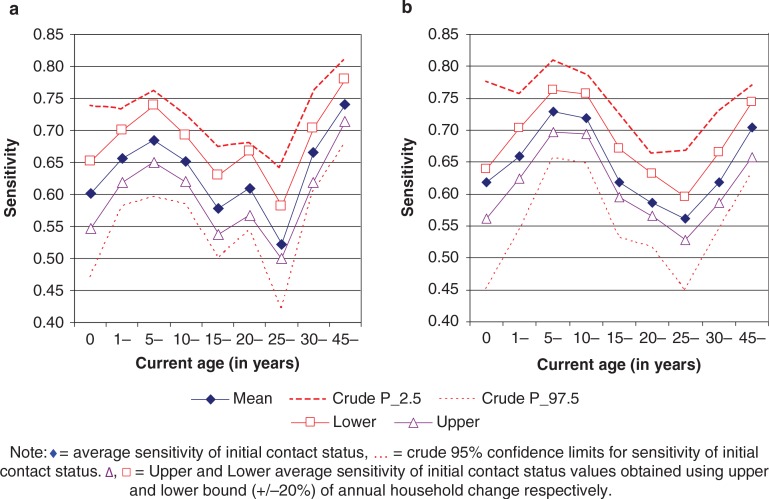
(a) Crude confidence intervals of initial contact status versus ‘true’ contact status (sensitivity) for females by age. (b) Crude confidence intervals of initial contact status versus ‘true’ contact status (sensitivity) for males, by age.

From Figs. 2a and 2b, we noted that the crude 95% confidence intervals for the sensitivity of contact status were much wider than those obtained using a relative precision of 20% (chosen arbitrarily) of the mean annual household change. The width was more pronounced for those aged less than 1 year and those aged 25–29 years.

The crude 95% confidence intervals gave the bounds within which the true value of age/sex specific sensitivity of initial contact status was expected to lie without varying the input parameters of the model. However, even after varying the estimates of change of household by up to 20%, we still got values of sensitivity of initial contact status that lay within the crude 95% confidence intervals across all age groups.

#### Backward sensitivity of initial contact status

Some incident cases that arise during a follow-up period of study may partly be attributable to contact which occurred prior to the period of study. Table 4 shows the expected trend of decline in sensitivity of initial contact status with increasing incubation period.

**Table 4:** Values of backward sensitivity of contact status (standard deviation) for contacts of all index cases from simulations with long incubation period after 50 simulation runs of a 10-year period with fixed 5 years of follow-up

	Length of incubation period (in years)						

	5		7		9	
			
Age group (in years)	Male	Female	Male	Female	Male	Female
0–9	0.60 (0.0308)	0.60 (0.0295)	0.52 (0.0374)	0.52 (0.0310)	0.48 (0.0318)	0.48 (0.0308)
10–14	0.62 (0.0358)	0.59 (0.0394)	0.54 (0.0413)	0.50 (0.0438)	0.51 (0.0478)	0.45 (0.0386)
15–19	0.61 (0.0291)	0.57 (0.0405)	0.53 (0.0401)	0.46 (0.0391)	0.47 (0.0405)	0.43 (0.0456)
20–24	0.60 (0.0367)	0.60 (0.0482)	0.50 (0.0505)	0.50 (0.0424)	0.43 (0.0421)	0.46 (0.0586)
25–29	0.57 (0.0468)	0.67 (0.0373)	0.47 (0.0471)	0.57 (0.0434)	0.40 (0.0565)	0.54 (0.0475)
30–44	0.64 (0.0427)	0.78 (0.0390)	0.56 (0.0426)	0.71 (0.0484)	0.51 (0.0529)	0.70 (0.0448)
45+	0.68 (0.0320)	0.67 (0.0327)	0.60 (0.0449)	0.61 (0.0350)	0.56 (0.0402)	0.57 (0.0410)

## Discussion

This paper has demonstrated some of the complicated household dynamic issues, which affect efforts to study contact-associated spread of infectious diseases. As shown by Pickles (2–4) at community level, real-time contact tracing, even in acute infections, has challenges of identifying source cases of infection, their contacts as well as secondary cases. This problem is greater in chronic infections with long incubation periods. Whereas in acute infections, it is safe to assume static contact networks, this assumption fails in chronic infections where contact networks are dynamic. Thus, without good quality data, estimation of infection transmission through household contacts is a challenge.

Furthermore, various mathematical or statistical models have been developed to investigate infection transmission through contact networks (5, 19, 21–23, 29, 36). Most of these have assumed static contact leading to the potential under-estimation of household contact associated risks. This paper is one such attempt to use dynamic household contact networks and to provide relevant background for future improvements where more reliable data are available. We were able to estimate movements of index cases and their contacts and contact status misclassification.

Despite the significance of the results for public health, the study was not without limitations. The major assumption made was that rates of deaths, migration, and movement between households are constant and random during the period of the simulation. However, in most societies, household change by a parent may imply particular changes for their children but such data were not available.

We also assumed that disease status in index cases is not misclassified, that distance between households does not affect infection and that the incubation period is fixed. It is known that incubation period, for example, of tuberculosis (37, 38), may depend on age at exposure and dose of the infectious agent. Because little is known on the distribution of the incubation period of leprosy, we assumed different fixed incubation periods.

Finally, this study only looked at tracing contact within households to investigate the traceable extent of misclassification. Household is one of the many contact points and contact outside the household such as at work, school, travel, and community gatherings were not considered in our simulations.

Not even a detailed stochastic simulation model such as this can capture all of the household changes. A model is by definition a simplification of reality and should not be over-interpreted. The complexity of the model can vary depending on time and the research question. In this paper, the objective was to enhance the understanding of the principles of household dynamics and how they affect household contact.

The propensity of an individual to change household has important implications for contact status misclassification. A low sensitivity of initial contact status measure is a reflection of a high rate of household change. This study has shown that the distribution of sensitivity values with age is inversely related to the rate of household change, which is low in children but high in young adults. The observed earlier lower sensitivity among young adult females compared to males is related to earlier household change for females largely due to early onset of marriage. There were no apparent sex differences in sensitivity values for children aged less than 5 years, as their movements are largely dependent on their parents or guardians.

The longer the duration of follow-up, the greater the misclassification of contact status due to increased household dynamics. The ‘forward’ and ‘backward’ perspectives in this paper describe an important distinction of contacts arising during the follow-up period and unobserved earlier relevant contact before the start of study. We observed that, in general, values for ‘backward’ sensitivity are lower than ‘forward’ sensitivity of initial contact status. Further, the longer the incubation period, not only are differences more pronounced but also the lower the ‘backward’ sensitivity. This shows that the likelihood for an actual earlier contact to go unrecognised can be great leading to high contact status misclassification. It is therefore important to appreciate that, when dealing with household contact-associated spread of diseases, such as leprosy and tuberculosis, much of the new disease observed during follow-up may well be attributable to unobserved earlier household contact.

The effect of household change may be reduced by conducting studies on infection transmission within households for shorter periods of follow-up and for only those areas where the household change rates are low, thus ensuring minimum contact status misclassification. However, where such conditions are not possible, estimations of misclassification values from this study are useful in obtaining reliable estimates of risk of disease associated with household contact in the same or similar population settings.

This study has shown the extent of household contact misclassification in a rural African setting in northern Malawi. The demographic trends and issues that drive mobility in various African countries are similar (39–45) and the findings are generally applicable regionally. However, availability of good quality data to derive parameters for modelling to generate reliable estimates of risk of disease is a challenge. Currently, there is an increase in longitudinal studies, especially demographic surveillance sites (19, 46–48), where such data is increasingly available for use in more appropriate dynamic contact networks modelling.
